# Functional variations of the *TLR4* gene in association with chronic obstructive pulmonary disease and pulmonary tuberculosis

**DOI:** 10.1186/s12890-019-0939-y

**Published:** 2019-10-22

**Authors:** Zhongqi Li, Xuhua Mao, Qiao Liu, Huan Song, Biyu He, Peiyi Shi, Qun Zhang, Xiaona Li, Jianming Wang

**Affiliations:** 1 Department of Epidemiology, Center for Global Health, School of Public Health, Nanjing Medical University, Nanjing, 211166 People’s Republic of China; 2 Department of Clinical Laboratory, Yixing People’s Hospital, Wuxi, 214200 People’s Republic of China; 3 Health Management Center, The First Affiliated Hospital of Nanjing Medical University, Nanjing, 210029 People’s Republic of China; 4 Key Laboratory of Infectious Diseases, School of Public Health, Nanjing Medical University, 211166 Nanjing, People’s Republic of China

**Keywords:** Chronic obstructive pulmonary disease, Pulmonary tuberculosis, Toll-like receptor 4, Single nucleotide polymorphism, Genetic

## Abstract

**Objective:**

Chronic obstructive pulmonary disease (COPD) and pulmonary tuberculosis (PTB) share a number of common risk factors, including innate immunity-related genetic factors. In the present study, we compared the role of genetic variations of the *TLR4* gene in susceptibility to COPD and PTB and illuminated the underlying molecular mechanism of functional single-nucleotide polymorphisms (SNPs).

**Methods:**

A population-based case control study was performed in a Chinese Han population and included 152 COPD cases, 1601 PTB cases and 1727 controls. Five SNPs in the *TLR4* gene (rs10759932, rs2737190, rs7873784, rs11536889, and rs10983755) were genotyped using TaqMan allelic discrimination technology. We estimated the effects of SNPs using the odds ratio (OR) together with 95% confidence interval (CI). Dual-luciferase reporter vectors expressing different genotypes of SNPs were constructed and transfected into the human HEK 293 T cell line to explore their effects on potential transcription activity.

**Results:**

After Bonferroni correction, the genetic polymorphisms of all five SNPs remained significantly associated with COPD, while rs10759932 and rs2737190 were also associated with PTB. Compared with rs10759932-TT, individuals carrying TC (OR: 0.42, 95% CI: 0.28–0.64) or CC (OR: 0.24, 95% CI: 0.09–0.63) had a significantly reduced risk of COPD. However, individuals carrying TC (OR: 1.28, 95% CI: 1.11–1.49) or CC (OR: 1.26, 95% CI: 0.98–1.62) had an increased risk of PTB. The OR (95% CI) for allele rs10759932-C was 0.45 (0.32–0.62) for COPD and 1.18 (1.07–1.32) for PTB. For rs2737190, heterozygous AG was related to a decreased risk of COPD (OR: 0.32, 95% CI: 0.21–0.49) and an increased risk of PTB (OR: 1.30, 95% CI: 1.11–1.52). The dual-luciferase reporter assay showed decreased transcription activity caused by rs10759932-C and rs2737190-G.

**Conclusion:**

Genetic polymorphisms of rs10759932 and rs2737190 in *TLR4* are significantly related to both COPD and PTB but with inverse effects. The altered transcription activity caused by mutations in these two loci may partly explain the observed relationship.

## Introduction

Both chronic obstructive pulmonary disease (COPD) and tuberculosis (TB) primarily affect the lungs and are major causes of morbidity and mortality worldwide [1]. COPD is characterized by persistent airflow limitation because of various combinations of obstructive bronchiolitis and emphysema [2, 3]. The airflow limitation is usually not completely reversible and is associated with an abnormal inflammatory response of the lungs exposed to noxious particles and gases [4]. In a current nationwide, cross-sectional survey, the estimated overall prevalence of COPD in China was 13.6% among adults over 40 years old [5]. The etiology of COPD is complicated, and tobacco smoking is considered one of the foremost risk factors [2–4, 6–8]. Other factors, such as pulmonary tuberculosis (PTB), air pollution and low body mass index (BMI), may also contribute to COPD [6, 7]. TB is a chronic infectious disease caused by *Mycobacterium tuberculosis* (MTB) infection, of which PTB is the most common type [9]. In 2017, there was an estimation of 10 million new cases and 1.3 million deaths due to TB worldwide, and the number of new TB cases in China was approximately 889 thousand, making it one of the top 30 countries with high TB burden (www.who.int).

COPD and TB have common risk factors, such as tobacco smoking, malnutrition, low socioeconomic status and dysregulation of host defense functions [1, 10]. Both COPD and TB are characterized by an abnormal innate immune response of alveolar macrophages exposed to both cigarette smoke and Toll-like receptor (TLR) stimulation [11, 12]. Since the first discovery of TLR4 in humans in late 1997, 13 types of TLRs (TLR1-TLR13) have been described in mammals [13]. As an important pattern recognition receptor in the natural immune system, TLR4 serves to discern pathogen-associated molecular pattern (PAMP) molecules or endogenous damage-associated molecular pattern (DAMP) molecules [14, 15]. TLR4 can trigger inflammatory responses by inducing the synthesis and release of proinflammatory cytokines and chemokines. Single nucleotide polymorphisms (SNPs) among various TLR genes have been identified, and their association with susceptibility/resistance to certain infections and other inflammatory diseases has been reported [13]. The interrelationship between COPD and TB is very complex [16]. Growing evidence appears to point towards a bidirectional relationship between these two lung diseases where each may act as an independent risk factor for the other [17]. It appears that the susceptibility of an individual to develop COPD and active TB involves a complex interaction between genetic and environmental factors [16].

Previous studies have reported genetic polymorphisms of *TLR4* in association with the risk of COPD and PTB [18, 19], but these two diseases were analyzed independently, and the function of genetic variations was not explored in these studies. In the present study, we compared the effects of genetic variations of the *TLR4* gene on the susceptibility to COPD and PTB in a Chinese Han population. Furthermore, we constructed dual-luciferase reporter vectors expressing different genotypes and transfected them into human HEK 293 T cell lines to analyze the potential function of SNPs on the promoter activity of the *TLR4* gene.

## Materials and methods

### Study population and data collection

A total of 152 newly diagnosed COPD cases and 1601 PTB cases were recruited from Jiangsu Province, China from 2011 to 2016. Both COPD and PTB patients included in this study were diagnosed according to the national guidelines. Respiratory function tests were performed for suspected COPD cases using a standard spirometer to measure forced expiratory volume in 1 s (FEV1) and forced vital capacity (FVC) [20]. The COPD was defined as an FEV1/FVC ratio below 70%. COPD cases with HIV/AIDS, immunosuppressive conditions, active TB, malignancy and other severe respiratory diseases were excluded. PTB cases were diagnosed by specialized doctors following the guidelines recommended by the China Ministry of Health, which were based on clinical symptoms and signs, chest X-ray examination, sputum smear tests or sputum culture (http://www.chinatb.org). PTB cases aged < 18 years, with HIV/AIDS, immunosuppressive conditions, malignancy and other severe respiratory diseases were excluded. All patients were genetically unrelated in the Chinese Han population. We also enrolled 1727 healthy controls who were residents who participated in the local community-based health examination program. They had no history of COPD, TB, HIV/AIDS, immunosuppressive conditions, malignancy or other severe respiratory diseases. After informed consent was obtained, questionnaires were used to collect demographic characteristics, smoking habits, drinking habits and respiratory disease histories. We obtained 3–5 ml venous blood samples from each individual for genetic analysis. This study was approved by the ethics committee of Nanjing Medical University.

### SNP selection and genotyping

Five SNPs in the *TLR4* gene (rs10759932, rs2737190, rs7873784, rs11536889, rs10983755) were selected for genotyping based on the following criteria: (1) a minor allele frequency ≥ 0.05 in the Chinese Han population; (2) a Hardy-Weinberg equilibrium test *P* ≥ 0.05; and (3) SNPs located in the functional areas, such as the 5′-UTR or 5′ near the gene, exon or 3′-UTR. The information of these five SNPs is listed in Additional file 1: Table S1. We genotyped the SNPs using the TaqMan allelic discrimination technology on a 384-well ABI 7900HT real-time PCR system (Applied Biosystems, Foster City, CA, USA). Amplification was performed in 0.5 ml tubes, which included 25 μl of premixed Taq version 2.0 (Takara, Kyoto, Japan), 1 μl of 20 μM forward and reverse primers and 100 ng of genomic DNA. The calling rate was over 98%. The primer and probe sequences for each SNP are listed in Additional file 2: Table S2.

### Construction of reporter vectors

**Fig. 1:**
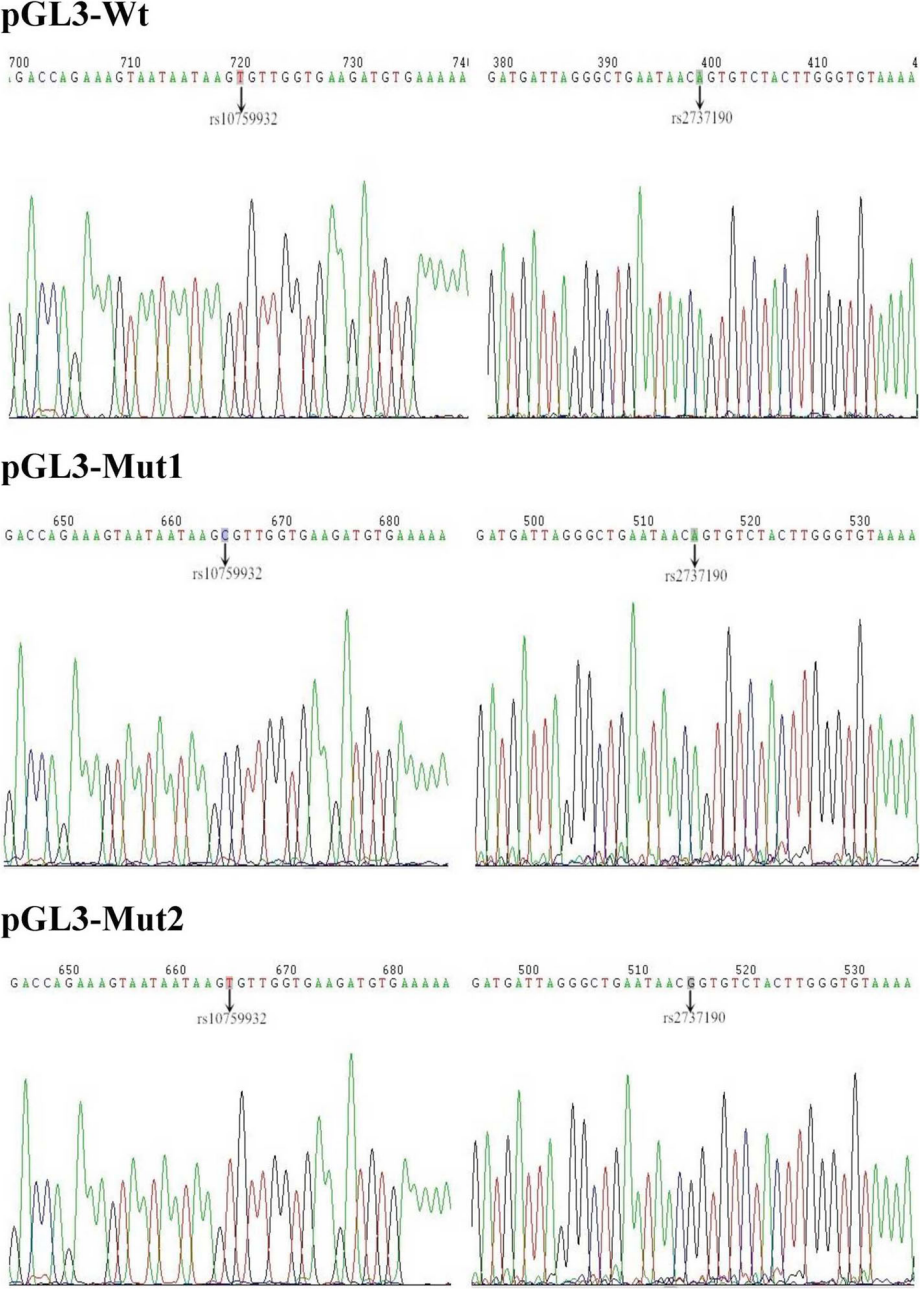
Sequencing of the pGL3 vector. pGL3-Wt: − 1309 T and − 2272 A; pGL3-Mut1: − 1309 C and − 2272 A; pGL3-Mut2: − 1309 T and − 2272 G

We obtained − 2588 to + 50 fragments of the *TLR4* gene containing rs10759932 (− 1309 T/C) and rs2737190 (− 2272 A/G) through GeneChem Corporation (Shanghai, China). They were named Wt (− 1309 T and − 2272 A), Mut1 (− 1309 C and − 2272 A) and Mut2 (− 1309 T and − 2272 G). PCR was performed to amplify the fragments of Wt, Mut1 and Mut2 in 0.5 ml tubes that included 0.5 μl of PrimeSTAR HS DNA polymerase (Takara), 1 μl of 10 μM forward and reverse primers, 4 μl of 2.5 mM each dNTP Mix (Takara), 10 ng of genomic DNA and 10 μl of 5 × PS Buffer (Takara). The forward primer was designed to contain the KpnI site (underlined): 5′-TTTCTCTATCGATA**GGTACC**TCGGCTATGGTGAAAACAACAG-3′. The reverse primer was designed to contain the XhoI site (underlined): 5′-CTTAGATCGCAGAT**CTCGAG**GAAGAAAACGCCTGCAGACCAGTG-3′. PCR products and pGL3-basic vectors (Promega, Wisconsin, USA) were double digested with KpnI and XhoI and were ligated with T4 DNA ligase (Takara) to construct dual-luciferase reporter vectors, which were named pGL3-Wt, pGL3-Mut1 and pGL3-Mut2, respectively. The sequencing of recombinant vectors was performed by GeneChem Corporation (Shanghai, China). Figure 1 illustrates the sequence validation of the recombinant vectors (pGL3-Wt, pGL3-Mut1 and pGL3-Mut2).

### Transfection and luciferase assays

Human HEK 293 T cells (GeneChem, Shanghai, China) in the logarithmic growth phase were used for cell suspensions, seeded in 24-well plates (the number of cells was approximately 10^5^), cultured in 37 °C with 5% CO_2_ and incubated until the fusion degree of cells reached approximately 60%. X-tremegene HP (Roche, Basel, Switzerland) was used for transfection. First, we transfected 1 μg of vectors and 2 μl of X-tremegene HP per well and dissolved X-tremegene HP and vectors in 100 μl opti-MEM (Gibco, Massachusetts, USA) according to this ratio and mixed and incubated them at room temperature for 20 min. Then, we aspirated 300 μl of the culture medium from the well plate, added 293 T cells to the mixture of vectors and X-tremegene HP, cultured the mixtures at 37 °C with 5% CO_2_, incubated them for 5–6 h and supplemented them with 200 μl complete medium containing 10% fetal bovine serum (Vian-Saga, Shanghai, China) to ensure that each culture well contained 500 μl medium. The pGL3-basic vector without an insert sequence was used as a negative control. Luciferase activity was detected 48 h after transfection. We used the Dual-Luciferase Reporter Assay System (Promega) to measure the activity of the *Firefly* luciferase gene and the *Renilla* luciferase gene. For each constructed vector, independent triplicate experiments were performed.

### Statistical analysis

Data were entered using EpiData 3.1 (EpiData Association, Denmark) and analyzed using SPSS version 25.0 (SPSS Inc., Illinois, USA). Student’s t-test (for continuous variables) and the *χ*^*2*^*-*test (for categorical variables) were used to analyze the differences in demographic variables and risk factors between cases and controls. The logistic regression model was used to estimate the effects of SNPs on susceptibility to COPD and PTB by calculating the odds ratio (OR) and 95% confidence interval (CI). We adjusted for age, sex, smoking and drinking to control for potential confounding factors. The significance level was defined as 0.05 in the present study. The Bonferroni correction was used to adjust the test level for multiple comparisons (0.05/5 = 0.01).

## Results

### General characteristics of study subjects

The basic characteristics of the cases and controls are listed in Table 1. The COPD case group consisted of 131 males and 21 females, and the PTB case group consisted of 1181 males and 420 females, while the control group consisted of 1272 males and 455 females. The distribution of ages was significantly different between the controls and the COPD group (*t* = 24.92, *P* < 0.001) or the PTB group (*t* = 4.84, *P* < 0.001). The sex distribution was different between the controls and the COPD group (*χ*^*2*^ = 11.60, *P* = 0.001). Tobacco smoking and alcohol drinking were also related to COPD or PTB (Table 1). Thus, we adjusted for age, sex, smoking and drinking when analyzing the effect of *TLR4* SNPs.

**Table 1:** Basic characteristics of cases and controls

Variables	Control (*n* = 1727)	COPD (*n* = 152)	PTB (*n* = 1601)	*t/χ* ^*2*^_COPD_	*P*_COPD_	*t/χ* ^*2*^_PTB_	*P*_PTB_
Age (years)							
Mean(±SD)	55.03(±17.70)	75.97(±8.93)	52.06(±17.71)	24.92	< 0.001^a^	4.84	< 0.001^a^
Sex [n(%)]				11.60	0.001^b^	0.01	0.941^b^
Male	1272 (73.7)	131 (86.2)	1181 (73.8)				
Female	455 (26.3)	21 (13.8)	420 (26.2)				
Smoking [n(%)]				34.88	< 0.001^b^	88.01	< 0.001^b^
Never	1101 (63.8)	60 (39.5)	762 (47.6)				
Ever	626 (36.2)	92 (60.5)	839 (52.4)				
Drinking [n(%)]				0.01	0.932^b^	8.94	0.003^b^
Never	1267 (73.4)	112 (73.7)	1246 (77.8)				
Ever	460 (26.6)	40 (26.3)	355 (22.2)				

^a^Student’s t-test; ^b^*χ*^*2*^ test

### Genetic variations of *TLR4* on the risk of COPD and PTB

Five SNPs were all in Hardy-Weinberg equilibrium in the control group (*P* = 0.12 for rs10759932, *P* = 0.07 for rs2737190, *P* = 0.57 for rs7873784, *P* = 0.94 for rs11536889, and *P* = 0.47 for rs10983755). As listed in Table 2, after the Bonferroni correction, the genetic polymorphisms of five SNPs were all significantly associated with the susceptibility to COPD, while rs10759932 and rs2737190 were also associated with the risk of PTB. Compared with rs10759932-TT, individuals carrying TC (OR: 0.42, 95% CI: 0.28–0.64) or CC (OR: 0.24, 95% CI: 0.09–0.63) had a significantly reduced risk of COPD. However, TC (OR: 1.28, 95% CI: 1.11–1.49) or CC (OR: 1.26, 95% CI: 0.98–1.62) carriers had an increased risk of PTB. The OR (95% CI) for allele rs10759932-C was 0.45 (0.32–0.62) for COPD and 1.18 (1.07–1.32) for PTB. For rs2737190, the heterozygous AG was related to a decreased risk of COPD (OR: 0.32, 95% CI: 0.21–0.49) and an increased risk of PTB (OR: 1.30, 95% CI: 1.11–1.52). However, the effect of the rs2737190-GG genotype was significant only for COPD.

**Table 2:** Genotype distributions of five SNPs between cases and controls

Gene	SNPs	Control, n(%)	COPD, n(%)	PTB, n(%)	*OR*_COPD_(95% *CI*)^a^	*P*_COPD_	*OR*_PTB_(95% *CI*)^a^	*P*_PTB_
*TLR4*	rs10759932^c^							
	TT	885 (51.6)	107 (72.8)	722 (45.7)	1		1	
	TC	675 (39.4)	35 (23.8)	697 (44.1)	0.42 (0.28–0.64)	< 0.001^b^	1.28 (1.11–1.49)	0.001^b^
	CC	154 (9.0)	5 (3.4)	161 (10.2)	0.24 (0.09–0.63)	0.004^b^	1.26 (0.98–1.62)	0.068
	T	2445 (71.3)	249 (84.7)	2141 (67.8)	1		1	
	C	983 (28.7)	45 (15.3)	1019 (32.2)	0.45 (0.32–0.62)	< 0.001^b^	1.18 (1.07–1.32)	0.002^b^
	rs2737190^c^							
	AA	630 (37.0)	91 (63.2)	518 (32.6)	1		1	
	AG	781 (45.9)	34 (23.6)	840 (52.9)	0.32 (0.21–0.49)	< 0.001^b^	1.30 (1.11–1.52)	0.001^b^
	GG	291 (17.1)	19 (13.2)	231 (14.5)	0.42 (0.24–0.72)	0.002^b^	0.94 (0.76–1.16)	0.563
	A	2041 (60.0)	216 (75.0)	1876 (59.0)	1		1	
	G	1363 (40.0)	72 (25.0)	1302 (41.0)	0.50 (0.38–0.66)	< 0.001^b^	1.04 (0.94–1.15)	0.443
	rs7873784^c^							
	GG	1426 (83.3)	105 (72.9)	1310 (83.1)	1		1	
	GC	275 (16.1)	33 (22.9)	256 (16.2)	1.55 (1.00–2.42)	0.052	1.02 (0.84–1.23)	0.860
	CC	11 (0.6)	6 (4.2)	11 (0.7)	16.72 (4.29–65.12)	< 0.001^b^	1.27 (0.54–3.00)	0.591
	G	3127 (91.3)	243 (84.4)	2876 (91.2)	1		1	
	C	297 (8.7)	45 (15.6)	278 (8.8)	1.95 (1.39–2.74)	< 0.001^b^	1.02 (0.86–1.21)	0.841
	rs11536889^c^							
	GG	1013 (59.0)	47 (31.1)	953 (60.2)	1		1	
	GC	611 (35.6)	67 (44.4)	535 (33.8)	2.68 (1.78–4.05)	< 0.001^b^	0.97 (0.84–1.13)	0.713
	CC	93 (5.4)	37 (24.5)	94 (5.9)	8.65 (5.04–14.82)	< 0.001^b^	1.10 (0.81–1.49)	0.559
	G	2637 (76.8)	161 (53.3)	2441 (77.1)	1		1	
	C	797 (23.2)	141 (46.7)	723 (22.9)	2.90 (2.28–3.68)	< 0.001^b^	0.98 (0.87–1.10)	0.730
	rs10983755^c^							
	GG	904 (52.5)	51 (33.8)	806 (50.7)	1		1	
	GA	679 (39.4)	70 (46.4)	644 (40.5)	1.90 (1.28–2.82)	0.002^b^	1.07 (0.92–1.24)	0.366
	AA	139 (8.1)	30 (19.9)	139 (8.7)	4.27 (2.49–7.31)	< 0.001^b^	1.09 (0.84–1.41)	0.533
	G	2487 (72.2)	172 (57.0)	2256 (71.0)	1		1	
	A	957 (27.8)	130 (43.0)	922 (29.0)	1.96 (1.55–2.50)	< 0.001^b^	1.06 (0.95–1.18)	0.270

^a^*OR* odds ratio, *CI* confidence interval; adjusted for age, sex, smoking and drinking; ^b^significant after the Bonferroni correction for multiple comparisons. ^c^with missing values

### Functional analysis of genetic variations

**Fig. 2:**
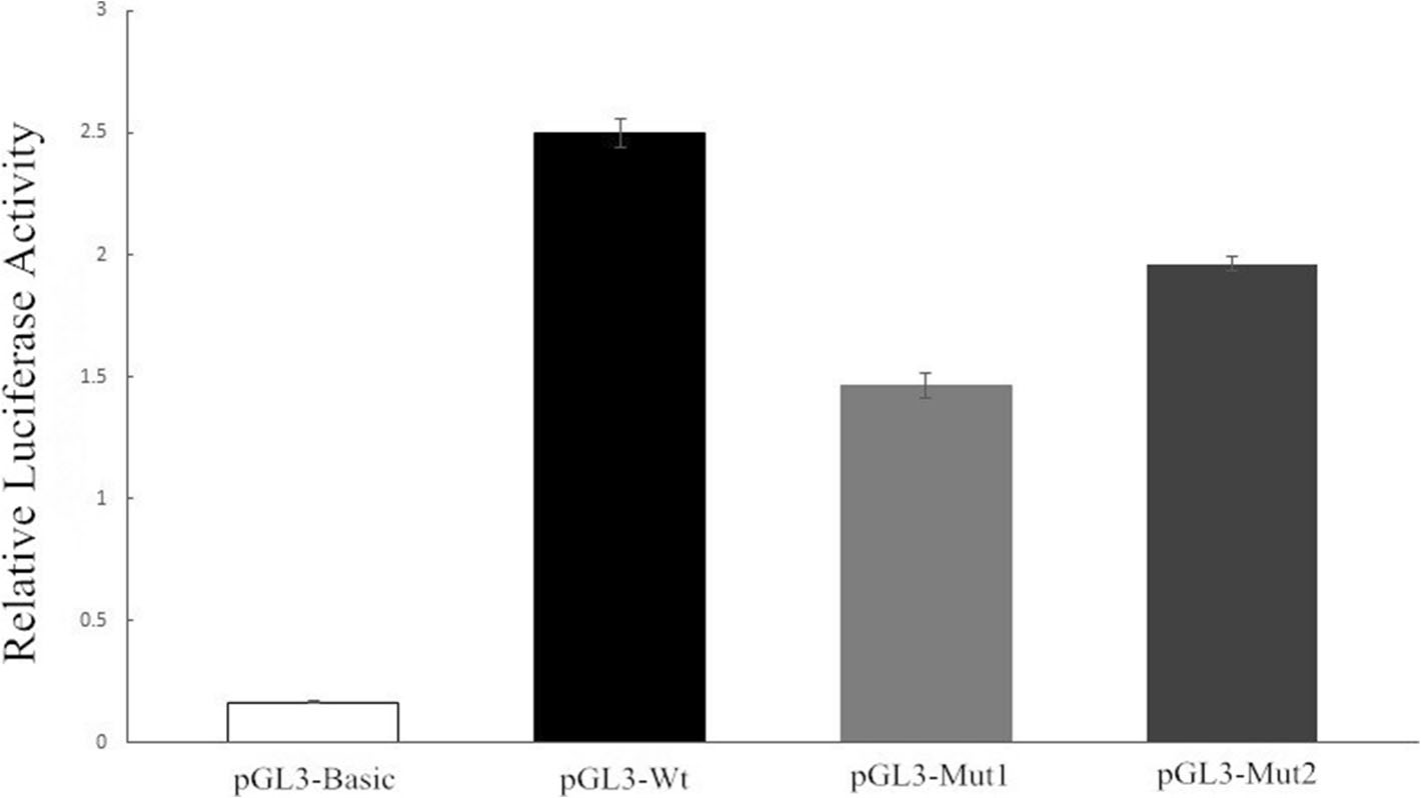
The relative luciferase activity of recombinant vectors. pGL3-basic: vector without an insert sequence; pGL3-Wt: − 1309 T and − 2272 A; pGL3-Mut1: − 1309 C and − 2272 A; pGL3-Mut2: − 1309 T and − 2272 G

As shown in Fig. 2, the mean (±SD) values of relative luciferase activity were 0.166 (±0.002) for pGL3-basic, 2.502 (±0.059) for pGL3-Wt, 1.466 (±0.051) for pGL3-Mut1 and 1.963 (±0.032) for pGL3-Mut2. Compared with pGL3-Wt, the relative luciferase activity was significantly reduced in both pGL3-Mut1 (*t* = 30.78, *P* = 0.001) and pGL3-Mut2 (*t* = 25.72, *P* = 0.001), indicating a potential role of genetic variations of *TLR4* rs10759932 (− 1309 T/C) and rs2737190 (− 2272 A/G) in regulating gene expression.

## Discussion

COPD is emerging as the third largest cause of human mortality worldwide, and there is growing evidence for comorbidity between COPD and TB, the leading cause of death globally due to respiratory infection [17]. COPD and TB share a number of common risk factors, including innate immunity-related genetic factors. In the present study, we compared the role of genetic variations of the *TLR4* gene in the susceptibility to COPD and PTB. We observed that the genetic polymorphisms of rs10759932 and rs2737190 were significantly associated with both COPD and PTB, but with inverse effects. Dual-luciferase reporter vectors suggested the potential function of these two SNPs regulating the transcription activity of the *TLR4* gene.

TLR4 is a pattern recognition receptor that participates in both innate and adaptive immunity. TLR4 is located on the surface of monocytes and macrophages and transmits activation signals after lipopolysaccharide (LPS) recognition, induces the expression of proinflammatory cytokines, such as IL-1, myeloid differentiation factor 88 (MyD88) and TNF-α, and forms receptor complexes, resulting in the activation of NF-κB and its translocation into the nucleus, which mediates inflammatory responses and adaptive immune responses [21]. TLR4 plays an important role in lung structure maintenance and smoking-induced emphysema and stimulates low-grade activation of the innate immune system, which is required for lung structural stability [22–24]. Previous studies have demonstrated that the expression of TLR4 on the surface of peripheral blood mononuclear cells and the concentrations of TNF-α, IL-6 and IL-8 in the serum of COPD patients were all significantly increased and positively correlated [25–27]. TLR4 influences the expression of inflammatory cytokines and chemokines, such as TNF-α, IL-1, IL-6 and IL-8, and these cytokines and chemokines interact with each other, which in turn regulate the expression of TLR4 in the airway of patients with COPD [25–27]. Speletas et al. investigated common TLR polymorphisms (*TLR2*-R753Q, *TLR4*-D299G and *TLR4*-T399I) in a group of heavy smokers (> 20 pack years). The presence of the *TLR4*-T399I polymorphism was associated with a 2.4-fold increased risk for COPD development, but not with the disease stage or frequency of exacerbations [28]. A study in Germany observed that the *TLR4*-D299G polymorphism was related to COPD [29]. In our study, five SNPs in the *TLR4* gene were all significantly associated with the risk of COPD, indicating its critical role in the development of COPD in the Chinese population.

It is estimated that approximately one third of the global population has been infected with MTB, but only 5–10% of these latently infected individuals eventually develop active TB, which may be attributed to different individual immunity [30, 31]. When MTB invades the host, TLR4 can recognize LPS, triggering the adaptor molecule MyD88 and other signaling pathways and initiating innate host defense against MTB [32, 33]. A study with mice found that mice with TLR4 were all alive at week 15 after intranasal inoculation with an MTB suspension, whereas 7 of 12 TLR4-deficient mice died [34]. The bacterial load of TLR4-deficient mice was 3-fold higher than that of mice with TLR4, and the levels of cytokines in TLR4-deficient mice were significantly decreased [34]. In the present study, we genotyped five functional SNPs in the *TLR4* gene, and only rs10759932 and rs2737190 were associated with PTB. Moreover, the effect of these two SNPs on the risk of COPD and PTB was inverse. This may be attributed to the dual role of the inflammatory response. On the one hand, inflammatory mediators directly or indirectly cause damage to tissues and cells. For example, the TLR4 signaling pathway mediates inflammation after ischemia-reperfusion and leads to tissue damage [35, 36]. On the other hand, it dilutes, kills and surrounds damage factors through inflammatory hyperemia and exudation reactions, and repairs and heals the damaged tissue through the regeneration of parenchymal and mesenchymal cells. Jiang et al. reported that the interaction between the endogenous matrix component hyaluronic acid and TLR4 can trigger an inflammatory response, maintain structural cellular integrity, and promote the recovery of tissue damage [37, 38].

In this study, we constructed dual-luciferase reporter vectors expressing different genotypes and transfected them into the human HEK 293 T cell line to explore the effects of SNPs on the *TLR4* gene. Our results showed that rs10759932-C (vs. T) and rs2737190-G (vs. A) can decrease the promoter activity of the *TLR4* gene. The mutations of these two loci not only weaken the capacity of the immune system to recognize MTB but also reduce the production and release of proinflammatory cytokines, resulting in an increased risk of PTB. In COPD, the excessive expression of proinflammatory cytokines damages lung tissue and cells beyond the tolerance of the body during the chronic inflammatory process, leading to various combinations of bronchitis, emphysema and pulmonary fibrosis, which in turn aggravate the condition of COPD patients. This may partly explain the reduced risk of COPD by mutations in these two loci.

There were several limitations in this study. First, we only analyzed five SNPs in the *TLR4* gene, and other immune-related genes were not considered. Second, although the dual-luciferase reporter vectors were used to estimate the altered transcriptional activity of the *TLR4* gene due to rs10759932-C and rs2737190-G, the affected transcription factors were unclear. The altered TLR4 protein and downstream cytokines need to be measured in future studies. Third, there is a complex interrelationship between COPD and PTB, one of which can serve as an independent risk factor for the other [16, 17]. We investigated the history of TB among COPD cases. However, we do not know the proportion of COPD among PTB cases because respiratory function tests were not routinely performed for suspected TB cases. Therefore, the concomitance of both diseases may have been an important confounding factor when we analyzed the effects of *TLR4* SNPs. Fourth, we only explored the functional effects of *TLR4* rs10759932 (− 1309 T/C) and rs2737190 (− 2272 A/G), which were related to both COPD and PTB. The effects of the other three SNPs on the transcriptional activity of *TLR4* were unclear.

In conclusion, genetic polymorphisms of rs10759932 and rs2737190 in *TLR4* are significantly related to both COPD and PTB but have inverse effects. The altered transcription activity caused by mutations in these two loci may partly explain the observed relationship. More work is necessary to identify specific causative variants underlying the observed associations. Further translational studies are warranted to delineate the molecular events behind these associations.

## Supplementary information

**Additional file 1 Table S1.** Information on five SNPs in the *TLR4* gene.

**Additional file 2 Table S2.** Primers and probes designed for genotyping.

## Data Availability

All data generated or analyzed during this study are included in this published article.

## Abbreviations

CI: Confidence interval
COPD: Chronic obstructive pulmonary disease
DAMPs: Damage-associated molecular pattern molecules
LPS: Lipopolysaccharide
MTB: *Mycobacterium tuberculosis*
MyD88: Myeloid differentiation factor 88
OR: Odds ratio
PAMPs: Pathogen-associated molecular pattern molecules
PTB: Pulmonary tuberculosis
SNP: Single nucleotide polymorphism
TB: Tuberculosis
TLR: Toll-like receptor
