# Supplementary material for: Functional variations of the TLR4 gene in association with chronic obstructive pulmonary disease and pulmonary tuberculosis
Source: BMC Pulm Med. 2019 Oct 22;19:184. doi: 10.1186/s12890-019-0939-y (PMC6805358; doi:10.1186/s12890-019-0939-y)
Supplement: Supplementary file 1 — Additional file 1 Table S1. Information on five SNPs in the TLR4 gene. [file 12890_2019_939_MOESM1_ESM.doc]

Table S1. Information on five SNPs in the *TLR4* gene.

| ID | Chromosome (GRCh38.p12) | Functional consequence |
| --- | --- | --- |
| rs10759932 | Chr9:117702866 | Upstream variant |
| rs2737190 | Chr9:117701903 | Upstream variant |
| rs7873784 | Chr9:117716658 | 3-prime untranslated region variant |
| rs11536889 | Chr9:117715853 | 3-prime untranslated region variant |
| rs10983755 | Chr9:117702392 | Upstream variant |
