# Supplementary material for: Functional variations of the TLR4 gene in association with chronic obstructive pulmonary disease and pulmonary tuberculosis
Source: BMC Pulm Med. 2019 Oct 22;19:184. doi: 10.1186/s12890-019-0939-y (PMC6805358; doi:10.1186/s12890-019-0939-y)
Supplement: Supplementary file 2 — Additional file 2 Table S2. Primers and probes designed for genotyping. [file 12890_2019_939_MOESM2_ESM.doc]

Table S2. Primers and probes designed for genotyping.

| Gene | SNPs | Primer (5’-3’) | Probe |
| --- | --- | --- | --- |
| *TLR4* | rs10759932 | F-CCCACAAATGGTGTACAGGAGTT | G: FAM-ATCTTCACCAACGCT-MGB |
|  | T>C | R-TGCAAGCTTCTGCTATGATTAAAAG | A: HEX-CATCTTCACCAACACT-MGB |
|  | rs2737190 | F-GGAGCATGCCTTATGCACACT | T: FAM-ACCCAAGTAGACACTGT-MGB |
|  | A>G | R-GACCTGTGATGATTAGGGCTGAA | C: HEX-ACCCAAGTAGACACCGT-MGB |
|  | rs7873784 | F-AGAACACTTAACATGAGAGGTACCC | C: FAM-TTCATTATACGAACTCTGC-MGB |
|  | C>G | R-GATGAATTAGCTCTAAAGATCAGCTGT | G: HEX-TTCATTATAGGAACTCTGC-MGB |
|  | rs11536889 | F-GTTGGGCAATGCTCCTTGA | G: FAM-ATTTTGGGAAGAGTGGAT-MGB |
|  | G>C | R-GAACCCCATTAATTCCAGACACA | C: HEX-CACATTTTGGGAACAGT-MGB |
|  | rs10983755 | F-ACCACAAAATGGTCCCTCACA | G: FAM-CTTGGTTTTTGACACGTT-MGB |
|  | G>A | R-TTCTACTGTAATATCCTCCAAGCACTTC | A: HEX-TTGGTTTTTGACACATTG-MGB |
